# Addressing the Global Neglect of Childhood Hearing Impairment in Developing Countries

**DOI:** 10.1371/journal.pmed.0040074

**Published:** 2007-04-03

**Authors:** Bolajoko O Olusanya

## Abstract

The number of children worldwide with hearing impairment is increasing, and these children face a number of social and educational obstacles.

The number of children worldwide with hearing impairment is increasing, and these children face a number of obstacles and burdens, given that spoken language is the predominant medium of communication and social interaction. Adequate auditory stimulation, in early childhood in particular, is the foundation for optimal speech and language development as well as the acquisition of literacy skills [1,2]. Failure to detect early and effectively manage within the first year of life a permanent hearing impairment that is congenital or that originates in the neonatal period has been associated with significant and irreversible deficits in speech and in linguistic, cognitive, and educational development [3,4].

In recognition of the growing and significant burden of hearing impairment globally, the World Health Assembly (WHA) in 1995 passed a resolution on the prevention and control of major causes of avoidable hearing impairment and on early detection in “babies, toddlers, and children” within the framework of primary health care [5]. The WHA is made up of ministers of health of United Nations member states and is responsible for determining the policies of the World Health Organization (WHO) to reflect the preferences of the UN member states [6].

Although neonatal hearing screening of all infants has become a standard of public health care in developed countries, the current priorities of major global health actors such as the World Bank and the United Nations Children's Fund (UNICEF) have yet to address the needs of infants with lifelong hearing impairment in developing countries [7,8]. Considering the substantial influence of global health priorities on government programs in many developing countries, this exclusion is likely to prevent vital public investment into early hearing detection services for an estimated 718,000 babies born annually in these countries [8,9]. This article therefore examines the current profile of global health-care financing and the opportunities for promoting this essential, time-bound health intervention in developing countries.

## The Global Burden of Childhood Hearing Impairment

The WHO global estimate for disabling hearing impairment (of a degree of severity >40 dBHL) has more than doubled from 120 million people in 1995 to at least 278 million in 2005 [10]. Two-thirds of individuals with hearing impairment live in developing countries, and hearing impairment in 68 million people is estimated to have originated from childhood [10]. About two to four babies per 1,000 live births are born annually in developed countries with permanent or sensorineural hearing impairment and this range may extend to six per 1,000 live births within the neonatal period in developing countries [9]. Permanent hearing impairment is an etiologically heterogeneous trait attributable to genetic and environmental causes, half of which are probably preventable [10].

Primary prevention—through immunization, health education, and improved maternal and child health services—is useful for preventing environmental causes of neonatal hearing impairment. But such prevention services have limited effectiveness in preventing the full spectrum of neonatal hearing impairment attributable to genetic or hereditary etiologies. Given the average life expectancy of 65 years in developing countries and 79 years in developed countries [8], lifelong hearing impairment is undoubtedly of great cost to society [11].

## Current Trends in Global Health Care Financing

Every government has a duty to provide health care for its population. In many developed countries health-care services (including universal newborn hearing screening [UNHS]) are freely provided, although some individuals may opt for private health services. The free provision of UNHS in many developed countries perhaps underpins the expectation that such screening should also be publicly funded in developing countries. But most governments are unable to wholly finance such programs, given the competing demands from prevailing communicable and fatal diseases, and thus have to seek external support. Early hearing detection from birth has been noted as an important and achievable goal for all countries [12]. But the failure to recognize childhood hearing impairment as a significant health condition for developing countries at the global level diminishes the prospects for publicly funded UNHS programs and may also divert attention away from the critical role of governments in facilitating private sector–led initiatives for the development of requisite services.

The World Bank's latest data on global spending for health care reveals striking variations in global health financing, as shown in Table 1 [13]. In 2002, public share of total health expenditures (a measure of how actively governments intervene to ensure the financing of public health services) ranged from 65% in high-income countries and 42% in middle-income countries to 29% in low-income countries. Global external funding accounted for 18% of total health expenditure in sub-Saharan Africa, 3% in South Asia, and 8% of total expenditure in low-income countries as a whole [13].

**Table 1:**
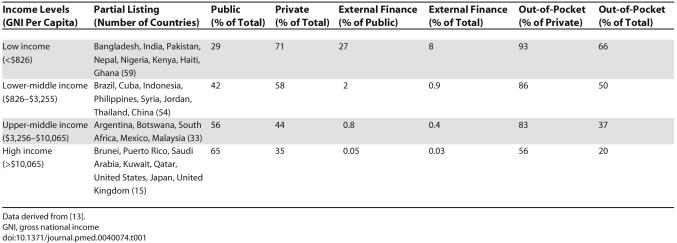
Global Health Expenditure Pattern by Income Levels

Moreover, user fees are commonly charged for most health-care services in developing countries. Such user fees result in out-of-pocket spending of 93% of private expenditure in low-income countries (more than 60% of total health expenditure) and about 85% in middle-income countries (40% of total health expenditure), compared to 56% in high-income countries (20% of total health expenditure). On a regional basis, the share of government contribution to health expenditure is lowest in South Asia, with private and out-of-pocket spending accounting for at least 75% (Figure 1). Although out-of-pocket spending is generally viewed as inequitable, it has remained the norm for health financing in most parts of the developing world and the situation is unlikely to change in the near future [13]. This trend therefore raises a fundamental question as to what extent local health-care priorities and services should be controlled by the policies of government or global health institutions.

**Figure 1:**
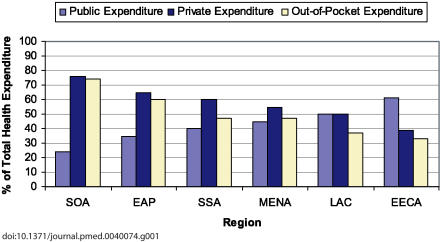
Global Health Expenditure Pattern by Region EAP, East Asia and Pacific; EECA, Eastern Europe and Central Asia; LAC, Latin America and the Caribbean; MENA, Middle East and North Africa; SOA, South of Asia; SSA, Sub-Saharan Africa

## Mechanisms for Financing Early Hearing Detection

Various levels of government determine public health priorities, taking into account the proportion of total revenue available for health care. Because resources are limited, public spending in low-income countries tends to be entirely concentrated on sustaining health systems rather than providing intervention services. As governments have to rely on additional funds from external sources, national priorities are therefore commonly guided (and sometimes overwhelmed) by diverse interests of multilateral institutions (World Bank, UNICEF, WHO, etc.) and donor partners despite their modest contributions to total health expenditure (Table 1) [14].

Given the substantial contributions of out-of-pocket spending to total national health expenditure, individual health preferences cannot be overlooked. Therefore, the key players in determining services to be provided are individual governments (national, state, or district), multilateral/donor agencies, and private providers, independently or in partnership. The factors that influence the priorities of these players and their relevance to services for early detection of childhood hearing impairment are now examined briefly.

### Government

Musgrove identified up to nine criteria for determining public expenditure, grouped into economic efficiency, ethical reasons, and political considerations as shown in Box 1 [15]. He argued that the decision as to which health services to spend public money on requires a systematic consideration of all nine criteria. But what constitutes partial or full satisfaction of these individual criteria, or what combination of partial satisfaction of the criteria would make screening justifiable was not stated explicitly. However, political considerations often have overriding influence over any technical considerations for the choice of services and the level of allocated public spending. Governments may favor services that are less costly rather than services that are more cost-effective because of the difficulty in accurately quantifying the benefits of services besides reduction in mortality.

Alternatively, the decision to embark on screening services, for instance, may be entirely predicated on the extent to which the conventional criteria for screening have been met [16], particularly when such costs can be (partly or fully) recovered through a financing scheme like health insurance or user fees. Sometimes the best screening option from a medical perspective is not the most cost-effective or the most favorable in economic terms. Pilot studies are valuable in deciding the actual tradeoffs that need to be made among the various considerations.

### Multilateral/donor organizations

The criteria for judging which interventions should be funded by donor agencies are often not explicit. However, the World Bank and WHO place emphasis on the burden of disease and on cost-effectiveness analyses of available interventions in setting health priorities [7].

Traditionally, burden of disease is measured by mortality or case fatality, which overlooks the full spectrum of the consequences of diseases on individuals or society. For instance, both communicable and chronic noncommunicable diseases are associated with significant physical and functional disabilities that are mostly lifelong. In order to address this conceptual limitation, a summary population measure such as disability-adjusted life years (DALYs) or its variant—quality-adjusted life years (QALYs)—is preferred. The World Bank and WHO maintain a database on the burden of disease for a variety of health conditions, which is used for cost-effectiveness analysis of various health interventions to establish priorities for developing countries [7]. This database is supplemented with available published evidence on the cost-effectiveness of interventions for diseases that are considered “important” by the UN agencies for global health.

However, this approach to global health financing may be biased against early childhood hearing impairment in developing countries for a number of reasons. Firstly, there is no data on DALYs associated with this category of hearing impairment. The Global Burden of Disease database only addresses hearing impairment of adult onset while treating childhood-onset hearing impairment as sequelae of other diseases. Secondly, a significant number of diseases and health conditions associated with early childhood hearing impairment were not considered in the report. Thirdly, without information on DALYs it is impracticable to evaluate the cost-effectiveness of available effective interventions to allow for comparison with other diseases contained in the Global Burden of Disease report. Fourthly, DALYs are difficult to compute where there is a scarcity of sound epidemiological data on the morbidity pattern of diseases, especially in developing countries. The use of DALYs has therefore been considered unsatisfactory for health planning in many developing countries and is rarely applied in practice [17,18].

**Box 1. Criteria for Prioritizing Health Expenditure**

**World Bank/WHO [7]**

- Burden of Disease
  ○ Mortality
  ○ DALYs
- Cost-Effectiveness Analysis
  ○ DALYs averted per unit cost

**Musgrove [15]**

**Economic**
  - Cost-Effectiveness
  - Public Goods
  - Externalities
  - Catastrophic Cost
**Ethical**
  - Horizontal Equity
  - Vertical Equity
  - Poverty
  - Rule of Rescue
**Political**
  - Public Demands

While measuring the cost-effectiveness of infant hearing screening compared to other child health interventions may be impracticable, the cost-effectiveness analysis of identifying a child with congenital hearing impairment can be reliably undertaken. Such an analysis would allow us to compare various intervention options such as targeted versus universal screening [19], one-stage versus multistage screening [20], or hospital-based versus community-based screening [21].

While cost-effectiveness analysis is currently the cornerstone of investment priorities in health care by the World Bank and its partners, the relevance of political, ethical, and cultural factors has also been acknowledged as equally important in allocating resources to specific diseases and interventions [7]. However, it would appear that WHA resolutions have little or no influence in this process despite their collective/representative legitimacy [22]. This trend needs to be reviewed to make the current prioritization approach more equitable and less contentious. In this regard it would be valuable to encourage and support all governments to undertake epidemiological studies using the existing WHO ear care survey protocol. Such baseline data can then serve as a guide for local systematic capacity-building for early detection and intervention services.

### Private and nongovernmental organizations

Private provision of services is often directed to satisfy identified needs of individuals and communities neglected by other service providers. However, the way individuals choose to order their spending priorities is often unpredictable and complex. For instance, in some communities socially stigmatized diseases may be considered more important than nonstigmatized diseases even if they are less prevalent or more costly to treat [23]. In a growing number of developing countries, including Brazil, China, Oman, Saudi Arabia, Iran, and the Philippines, newborn hearing screening services are offered for a fee in private and public hospitals under various small pilot programs. The emerging evidence accords with the prevailing pattern of health-care spending in general, which suggests that the value attached to health services cannot be judged solely by the level of public funding available for such services. In fact, it is not unusual to find that some indigent parents view helping their disadvantaged children as a vital investment towards their own future financial security and are thus willing to borrow or make the utmost financial sacrifices to obtain the needed services. Health priorities must therefore reflect important sociocultural factors relevant to the target population and the willingness of individuals to take advantage of effective time-bound health interventions from their personal albeit limited resources.

### Public–private partnership

The inability of governments to cater for diverse health needs with limited budgets has led to a growing trend towards local public–private partnerships for health-care delivery in low- and middle-income countries, especially for the most vulnerable populations [24,25]. For instance, a global partnership under the aegis of WWHearing (Worldwide Hearing Care for Developing Countries) is currently working with WHO towards the provision of affordable hearing aids and services to governments in developing countries (see http://www.who.int/pbd/deafness/activities/WWHearing/en/index.html). In addition, notable charitable organizations such as Christoffel-Blindenmission, Lions Club International, and Rotary International already have networks for supporting individuals with hearing impairment in many developing countries, which can be channeled towards early hearing detection services. In such partnerships, the role of government is geared towards creating an enabling environment for private participation at various levels. This includes policy formulation in line with best practices, monitoring and control, and provision of fiscal incentives as well as human resource training and development. Public education on preventable causes of hearing impairment, such as ototoxic drugs and consanguineous unions, is also feasible and inexpensive. Private participation may also take place through the activities of local nongovernmental organizations or commercial service providers. Most nongovernmental organizations operate on a not-for-profit basis but may charge modest fees to cover operational costs. Partnerships are most valuable and thrive where traditional ways of working independently have a limited impact on a problem and where core competencies or expertise of the parties are complementary [24].

Emerging evidence from ongoing pilot programs in countries such as Nigeria, South Africa, Malaysia, Brazil, and Poland demonstrate the effectiveness of different models of service delivery through public–private partnerships. However, the sustainability of these initiatives may be undermined by the continued lack of public sector support and the overwhelming preoccupation with fatal diseases by major actors in global health. Since infant hearing screening is now routinely provided in developed countries, failure to extend such a program to developing countries where about 90% of children with permanent hearing impairment live will only exacerbate health inequalities between the rich and poor nations of the world. WHO also acknowledges that it is important to ensure that screening interventions aimed at diseases in developed countries should be made available to developing countries where these conditions have emerged as important health problems [26].

## Ethical Considerations for Early Hearing Detection

The moral imperatives for providing early hearing intervention services, even in the absence of data on the cost-effectiveness of available interventions relative to other diseases, have been discussed in greater detail elsewhere [27]. A few points are worth restating.

Firstly, the effectiveness of interventions for optimal outcomes in speech, language, and cognitive skills is limited to the first year of life [3,4]. Secondly, if an apparently well child has a hidden abnormality, detectable at birth, that will most likely become obvious at an age when the prospects for effective intervention will have significantly diminished, parents will want to have this information as soon as it is practically possible [28–30]. Thirdly, declaring a child with congenital hearing impairment as “well and normal” at discharge after routine neonatal examination without simple and quick hearing screening tests can be morally disturbing for health-care professionals, especially when parents are willing to pay for such services if required. The new UN Convention on the rights of persons with disabilities should provide a further moral impetus for all to act [31].

Some have argued that if cost-effectiveness analysis is to have a larger role in health-care policy, an attempt must be made either to convince the public of its often overriding influence or to balance a consideration of its merits with a greater concern for equity [32]. Every government owes its citizens the right to be educated on best practices in health-care delivery, regardless of the limitations of public funding to deliver such services. This is a moral obligation. From this standpoint, parents should be educated on the current possibilities in early detection and intervention for early childhood hearing impairment regardless of the parents' financial status or the government's ability to provide the requisite services. Additionally, while it is unethical to withhold infant hearing screening in places where rehabilitation services are poor, it is also ethically questionable to continue screening without any systematic effort to improve relevant intervention services, including community support for individuals with hearing impairment.

## Conclusions

Current global health priorities for developing countries have yet to pay attention to the WHA resolution on hearing impairment, which acknowledges this condition in early childhood as a significant health problem. The prospects of any immediate action are uncertain, since vital data required by the current approaches to global disease prioritization are scarce and of limited value where available. Although external assistance constitutes a small proportion of total health expenditure in low- and middle-income countries, the priorities of multilateral/donor organizations still have a great influence on national health priorities and public sector–led systematic investment in requisite capacity-building for early hearing detection and intervention services. There is an urgent need therefore to stimulate public–private partnerships to preserve the collective/representative legitimacy of WHA resolutions, recognize individual health preferences through out-of-pocket spending, and ensure that those willing to take advantage of time-bound interventions for permanent and early-onset hearing impairment are not unduly disenfranchised by its current omission in the global health agenda for developing countries.

## Abbreviations

DALY: disability-adjusted life year
UN: United Nations
UNHS: universal newborn hearing screening
UNICEF: United Nations Children's Fund
WHA: World Health Assembly
WHO: World Health Organization
