# Correction: Addressing the Global Neglect of Childhood Hearing Impairment in Developing Countries

**DOI:** 10.1371/journal.pmed.0040203

**Published:** 2007-06-26

**Authors:** Bolajoko O Olusanya

In *PLoS Medicine*, volume 4, issue 4, doi:10.1371/journal.pmed.0040074:

The author inadvertently omitted the following citation that addresses the same topic (at the time of publication of the *PLoS Medicine* article, the article in *The Lancet* was still in press):

Olusanya BO, Newton VE (2007) Global burden of childhood hearing impairment and disease control priorities for developing countries. Lancet 369: 1314–1317.

The author did, however, cite this *Lancet* paper, as reference [10], in an earlier Correspondence article published in *PLoS Medicine*: Olusanya BO (2007) “The Right Stuff”: The Global Burden of Disease. PLoS Med 4(2): e84. doi:10.1371/journal.pmed.0040084

